# Discovery of a Non-Peptidic Inhibitor of West Nile Virus NS3 Protease by High-Throughput Docking

**DOI:** 10.1371/journal.pntd.0000356

**Published:** 2009-01-13

**Authors:** Dariusz Ekonomiuk, Xun-Cheng Su, Kiyoshi Ozawa, Christophe Bodenreider, Siew Pheng Lim, Zheng Yin, Thomas H. Keller, David Beer, Viral Patel, Gottfried Otting, Amedeo Caflisch, Danzhi Huang

**Affiliations:** 1 Department of Biochemistry, University of Zürich, Zürich, Switzerland; 2 Research School of Chemistry, The Australian National University, Canberra, Australia; 3 Novartis Institute for Tropical Diseases, Chromos, Singapore; Wyeth Research, United States of America

## Abstract

**Background:**

The non-structural 3 protease (NS3pro) is an essential flaviviral enzyme and therefore one of the most promising targets for drug development against West Nile virus (WNV) and dengue infections.

**Methodology:**

In this work, a small-molecule inhibitor of the WNV NS3pro has been identified by automatic fragment-based docking of about 12000 compounds and testing by nuclear magnetic resonance (NMR) spectroscopy of only 22 molecules. Specific binding of the inhibitor into the active site of NS3pro and its binding mode are confirmed by ^15^N-HSQC NMR spectra. The inhibitory activity is further validated by an enzymatic assay and a tryptophan fluorescence quenching assay.

**Conclusion:**

The inhibitor [4-(carbamimidoylsulfanylmethyl)-2,5-dimethylphenyl]-methylsulfanylmethanimidamide has a good ratio of binding affinity versus molecular weight (ligand efficiency of 0.33 kcal/mol per non-hydrogen atom), and thus has good potential as lead compound for further development to combat West Nile virus infections.

## Author Summary

An estimated 2.5 billion people are at risk of diseases caused by dengue and West Nile virus. As of today, there are neither vaccines to prevent nor drugs to cure the severe infections caused by these viruses. The NS3 protease is one of the most promising targets for drug development against West Nile virus because it is an essential enzyme for viral replication and because success has been demonstrated with the closely related hepatitis C virus protease. We have discovered a small molecule that inhibits the NS3 protease of West Nile virus by computer-aided high-throughput docking, and validated it using three experimental techniques. The inhibitor has potential to be developed to a drug candidate to combat West Nile virus infections.

## Introduction

West Nile virus (WNV) and the closely related dengue virus, members of the family *Flaviviridae*, are worldwide-spread global threats transmitted by mosquito bites. WNV encephalitis infects mainly birds but also other vertebrates including humans. It has been reported in four continents during the last decade [1] and has been spreading in North America causing several thousand cases per year since 1999 [2]. Despite its growing distribution and epidemic character, there are no specific antiviral treatments that can prevent or cure this infection.

The non-structural 3 protease (NS3pro) is one of the most promising targets for drug development against flaviviridae infections because it is responsible for cleavage of the viral polyprotein precursor and plays a pivotal role in viral replication [3],[4]. Moreover, two inhibitors of the closely related hepatitis C virus protease are under late-stage development [5],[6],[7],[8]. Site directed mutagenesis focused on the NS3pro cleavage sites in the polyprotein precursor abolishes viral infectivity [4]. Furthermore, the activity of NS3pro is significantly increased by the presence of a 47-residue region of the non-structural cofactor 2B (NS2B) [9]. There are two X-ray structures of WNV NS2B-NS3pro (the term ‘protease’ will be used in the following to simplify notation) in complex with inhibitors: one with the substrate-based tetrapeptide benzoyl-norleucine-lysine-arginine-arginine-aldehyde (Bz-Nle-Lys-Arg-Arg-H, PDB code 2fp7 [10]) and the other with bovine pancreatic trypsin inhibitor (BPTI, PDB code 2ijo [11]). The two X-ray structures with different inhibitors have similar conformations of the protease and are therefore appropriate for structure-based drug design. (Note that only the complex with the tetrapeptide inhibitor was available when the fragment-based docking was performed). The protease adopts a chymotrypsin-like fold with two six-stranded *β*-barrels. The binding pocket is small and very shallow with the catalytic triad (His51-Asp75-Ser135) located at the cleft between the two *β*-barrels.

The WNV protease is a very difficult target as is witnessed by the very small number of known non-peptidic inhibitors. Furthermore, only few molecules emerged as inhibitors of the WNV protease (in the micromolar range) from a library of more than one million compounds submitted to a high-throughput *in vitro* screening campaign [12]. Recently published efforts on inhibitor development against flaviviral proteases focused mostly on peptidomimetics [13],[14],[15],[16] and only few non-peptidic compounds have been reported [17],[18],[19] leaving open space for further investigation aimed at viral chemotherapy. The preferred amino acids at the nonprime part of the protease active site are arginine at the P1 position and arginine or lysine at the P2 position [4] underlining the role of electrostatic interactions with the negatively charged S1 and S2 pockets. Most of the reported active compounds have charged moieties, with the guanidino group being the most frequent. They include a class of D-arginine based 9–12 mer peptides [14], peptide aldehyde inhibitors [13],[16], and five non-peptidic guanidino compounds reported by Ganesh et al. [17]. Non-charged inhibitors include a series of 8-hydroxyquinoline [19], some uncompetitive inhibitors [18], and 15 inhibitors reported in PubChem BioAssay database [20]. Inhibitors for the close related NS3 proteases of Hepatitis C, dengue, and yellow fever virus have also been identified [8],[21],[22],[23],[24],[25],[26],[27],[28],[29],[30],[31],[32],[33],[34],[35]. In this paper, we report the discovery of a WNV protease inhibitor by our *in silico* high-throughput screening approach and experimental validations.

## Methods

The *in silico* screening was performed by a fragment-based docking procedure and an efficient evaluation of binding free energy with electrostatic solvation. All of the calculations were performed on the WNV protease from its complex with the tetrapeptide inhibitor Bz-Nle-Lys-Arg-Arg-H (PDB code 2fp7 [10]).

### Evaluation of Binding Free Energy with LIECE

The linear interaction energy with continuum electrostatics (LIECE) approach was introduced and tested first on aspartic proteases [36] and recently further validated on kinases [37]. Here, only a brief overview of the method is presented, while the development of the LIECE model for the WNV protease is presented in the section Results and Discussion. The essential idea of linear interaction energy models is that the free energy of binding can be calculated by considering only the end points of the thermodynamic cycle of ligand binding, i.e., bound and free states. For this purpose, one usually calculates average values of interaction energies from molecular dynamics (MD) simulations of the isolated ligand and the ligand/protein complex [38],[39]. In this way, the free energy of binding can be approximated by
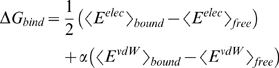
(1)where 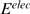 and 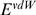 are the electrostatic and van der Waals interaction energies between the ligand and its surroundings. The surroundings are either the solvent (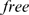) or the solvated protein (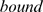), and the 〈 〉 denotes an ensemble average sampled usually by explicit water MD simulations. We have suggested that it is possible to avoid the MD sampling by replacing it with a simple energy minimization, and postprocessing of the minimized structures by a rigorous treatment of solvation within the continuum electrostatics approximation [36]. The LIECE approach is very efficient and has a predictive accuracy of about 1.0 kcal/mol for 13 and 29 peptidic inhibitors of BACE-1 and HIV-1 protease, respectively [36]. Similar accuracy has been reported recently for five kinases [37].

### Preparation of the Protease Structure

The coordinates of WNV protease in the complex with the tetrapeptide aldehyde inhibitor Bz-Nle-Lys-Arg-Arg-H were downloaded from the PDB database (PDB entry 2fp7 [10]). All water molecules were removed. The spurious termini at the segment missing in the X-ray structure (residues 28–32 in chain B) were neutralized by the −COCH_3_ group and the −NHCH_3_ group at the N-terminus and C-terminus, respectively. The 37 peptidic inhibitors used in this study include Bz-Nle-Lys-Arg-Arg-H (IC_50_ = 4.1 *µ*M) and a series of 36 related inhibitors with an aldehyde warhead and two or three positively charged side chains (IC_50_ values ranging from 0.4 to 463 *µ*M, see Text S1) synthesized in the same laboratory and tested all with the same enzymatic assay [13]. The initial binding conformations were modeled manually according to the binding mode of Bz-Nle-Lys-Arg-Arg-H because all inhibitors have similar backbone structure and are covalently bound to the Ser135 side chain by an ester linkage.

For a covalently bound inhibitor, the binding energy can be decomposed into 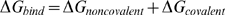. Noncovalent interaction energies in covalent complexes had been investigated before [40],[41]. In the case of irreversible inhibitors, 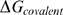 is determinant, and therefore improving the noncovalent complementarity within a covalently bound complex does not necessarily improve the potency of an inhibitor [40]. For reversible inhibitors, e.g., the 37 peptidic inhibitors used in this study [13], 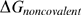 and 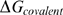 are both important for binding. Since the peptidic inhibitors have similar backbone structure and there is no large conformational change of the protein upon minimization, we assume that the energy contributions from 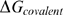 is similar for all inhibitors and the differences in binding affinities originate from 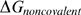. Therefore, for interaction energy calculations, the atoms involved in the ester bond between protein and inhibitor were neglected as well as the adjacent -OH group of Ser135 and -C(H)OH group of inhibitor to avoid steric clashes. The resulting empty valencies on protein and inhibitors were filled with hydrogen atoms.

### Preparation of the Compound Libraries

The compounds were selected by applying strict filtering criteria from the iResearch database (ChemNavigator Inc., 2006) using Filter v2.0.1 (OpenEye Scientific Software, Inc.). The selection of compounds was based on the property of the active site pockets S1 to S3, which contain 19 hydrogen bond acceptors. Therefore, compounds with multiple hydrogen bond donors are expected to have higher chances to bind. Furthermore, there are five aspartate side chains in (or very close to) the S1–S3 pockets, and most of the previously discovered peptidic and nonpeptidic inhibitors have at least one positive charge [12],[13]. Therefore, to generate a focussed sublibrary, at least five hydrogen bond donors or one positive charge was used as selection criteria. From a library of over 6 million molecules, only 11715 compounds met one of the two criteria. Of these 11715 compounds, 5882 are neutral, 4198 have one positive charge, 1503 have more than one positive charge, and the remaining 132 compounds have negative charge(s). Final preparation included the assignment of CHARMm atom types, force field parameters [42], and partial charges [43],[44], and energy minimization with a distance dependent dielectric function using the program CHARMM [45].

### High-throughput Docking

The fragment-based docking of the database (of prefiltered compounds) consists of four consecutive steps which have been detailed in a previous work [46]. Briefly, the four steps are: (1) Decomposition of each molecule of the library into mainly rigid fragments by the program DAIM [47], (2) fragment docking with evaluation of electrostatic solvation [48],[49] by the program SEED [50],[51], (3) flexible docking of each molecule of the library using the position and orientation of its fragments as anchors by the program FFLD [52],[53], and (4) final minimization by CHARMM [45] using the CHARMm force field [42] followed by evaluation of the LIECE binding free energy for the best poses. The protein structure was kept rigid in all steps. Furthermore, in all continuum electrostatic calculations (in SEED and LIECE) the interior dielectric constant was set to 2.0 to partially account for the electronic polarizability and dipolar reorientation effects of the solute [54]. The advantage of the multi-step approach used in this and previous *in silico* high-throughput screening campaigns [37],[46],[55] is that intermediate results (e.g., binding modes of most favorable fragments) can be used to focus the screening [56].

### Computational Requirements

The *in silico* screening of the nearly 12000 compounds, i.e., docking and binding energy evaluation, took about 30 hours on a Beowulf cluster of 100 Opteron 1.8 GHz CPUs. The LIECE approach requires 4 minutes (mainly for the finite-difference Poisson calculations with grid spacing of 0.4 Å) of a single Opteron 1.8 GHz CPU for each pose.

### Enzymatic assay

The enzymatic assay was performed as described in Knox et al. [13]. Briefly, compounds were mixed with 50 nM West Nile CF40-gly-NS3pro187 and incubated at 37°C for 30 min in 50 mM Tris, pH 8.5 and 20% glycerol, followed by addition of 20 mM substrate, Bz-nKRR-AMC. The fluorophore, 7-amino-4-methylcoumarin (AMC) group at the C-terminus becomes fluorescent when cleaved from the peptide substrate. Reaction progress was monitored continuously at 37°C (excitation 385 nm, emission 465 nm) in a Victor3V plate reader (Perkin Elmer) as an increase in fluorescence (RFU/min) which was then converted to Ms^−1^ from a standard AMC calibration curve. Percentage of inhibition of enzyme activity was determined by comparing the initial velocities in the enzyme reaction in presence of increasing concentration of compound **1** (from 0 to 500 mM) against control wells without compounds. IC_50_ values were derived for inhibitors by fitting the calculated initial velocities to a non linear regression curve using GraphPad Prism software. Each point of the IC_50_ curve was carried out in duplicate during a single experiment.

### Tryptophan fluorescence assay

Binding of compound **1** was measured by a competition assay with an noncovalently bound inhibitor (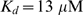, see Text S1) that quenched tryptophan fluorescence upon binding in the protease active site [12]. 3 *µ*M of protein were titrated by this competitive inhibitor (from 0 to 40 *µ*M) in the absence or presence of varying concentrations of compound **1** (70, 140 and 210 *µ*M) in Tris 50 mM, pH 7.5, and NaCl 50 mM. 90 *µ*l of each dilution were thereafter transferred to a UV-star Greiner 96-wells microplate. After 1 h incubation at room temperature, fluorescence was measured at 25°C on a Tecan Safire2 with 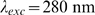 (bandwidth 10 nm) and 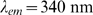 (bandwidth 20 nm). 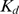 for compound **1** was inferred from the effect of the varying concentrations on the 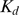 of the reference compound as described [57].

### NMR Validation

NMR spectroscopy analysis (^1^H chemical shifts and ^15^N-HSQC spectra) was performed as described elsewhere [58]. The Lys96 to Ala single-point mutant of the NS2B cofactor (NS2B(K96A)) was used because it significantly reduced self-cleavage, preventing gradual build-up of sample heterogeneities [59]. The binding affinity of compound **1** to the protease was determined by monitoring the chemical shifts of ^15^N-HSQC cross peaks as a function of ligand-to-protein concentration ratio. The curves of different cross peaks were fitted using the following equation which assumes 1∶1 binding stoichiometry
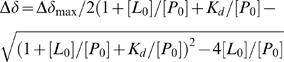
where 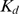 is the dissociation constant, 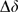 and 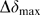 are the observed and maximal chemical shift change, and [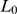] and [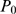] are the total ligand and protein concentrations, respectively. Fitting was performed using the non-linear fitting option in Origin 6.0 (Microcal, Northampton, MA, USA) [60],[61].

## Results/Discussion

### Validation of the LIECE model on the WNV protease

Experimental IC_50_ values were first converted into binding free energy by 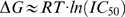, where relative values of IC_50_ are assumed to parallel relative free energies of binding in a closely related series of inhibitors [62],[63]. The equations used for fitting the interaction energy terms to the experimental free energy of binding are a two-parameter model with continuum electrostatics [36],[37]

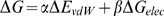
(2)and a three-parameter model with decomposed electrostatics
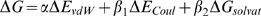
(3)where 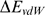 is the intermolecular van der Waals energy and 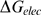 is the sum of two terms: the intermolecular Coulombic energy *in vacuo* 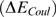 plus the change in solvation energy of inhibitor and protein upon binding 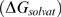. Additional models were tested by taking into account the loss of translational and rotational degrees of freedom upon binding and the freezing of rotatable bonds of the inhibitor. No improvement was observed for the 37 peptide aldehyde inhibitors of WNV protease used in this study.

**Figure 1:**
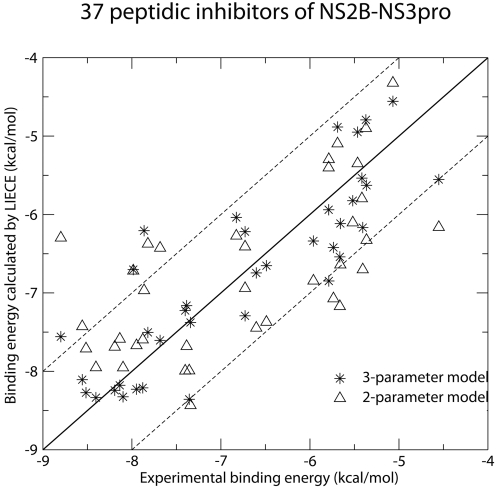
Comparison of the calculated versus experimental binding free energies for 37 peptidic inhibitors of the WNV protease [13]. Peptide sequences are listed in Table S-I (found in Text S1). The solid diagonal is the ideal line of perfect prediction. The dashed diagonals delimit the 1 kcal/mol error region.

The parameters obtained by least-squares fitting are given in Table 1 and the scatter plot of binding energies versus experimental binding energies is shown in Figure 1. The three-parameter seems to better fit the experimental data than the two-parameter model as evidenced by a smaller value in the root mean square of the error in the energy (0.63 kcal/mol vs. 0.93 kcal/mol), and larger cross-validated 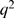 (0.66 vs. 0.34). Therefore, the three-parameter model was selected as scoring function to prioritize the poses obtained by high-throughput docking. Note that in this study, 37 peptide aldehyde inhibitors with either two or three positive charges were used to derive the LIECE model. Adding neutral inhibitors or inhibitors with only one formal charge for the fitting leads to worse results because of the large difference in polarization. For such inhibitors with broad range of formal charges (from 0 to 3), semiempirical quantum mechanical calculations can be used to evaluate polarization effects (an approach termed QMLIECE [64]), but the present docking campaign and ranking were performed before the development of QMLIECE.

**Table 1:** The two-parameter and three-parameter LIECE models derived using 37 peptide aldehyde inhibitors of WNV protease.

	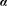	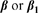	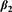	Error^a^ [kcal/mol]	LOO^b^ cv 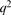
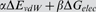	0.161(7)^c^	0.075(11)	-	0.93	0.34
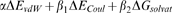	0.078(21)	0.051(12)	0.045(13)	0.63	0.66

Peptide sequences are listed in Table S-I (found in Text S1).

a Root mean square of the difference between 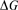 values predicted by LIECE and measured *in vitro*.

b Leave-one-out cross-validated 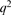.

c The standard deviation of the parameters from the leave-one-out procedure is in parentheses, e.g., 0.075(11) abbreviates 0.075±0.011.

### *In Silico* Screening

**Figure 2:**
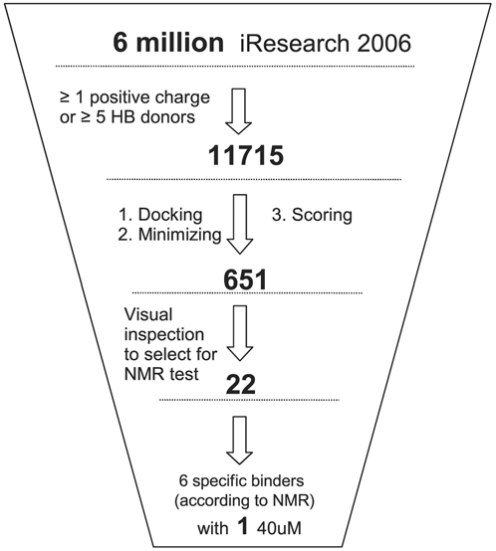
Schematic picture of the *in silico* screening campaign. Docking of the 11715 compounds with one more positive charges or at least five hydrogen bond donors was performed by DAIM/SEED/FFLD using the 2fp7 structure of the WNV protease as explained in the text.

**Figure 3:**
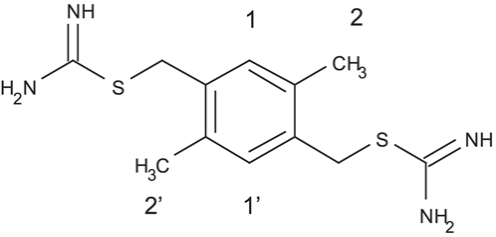
Compound 1, 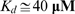 according to ^1^H chemical shifts (see Figure 4).

**Figure 4:**
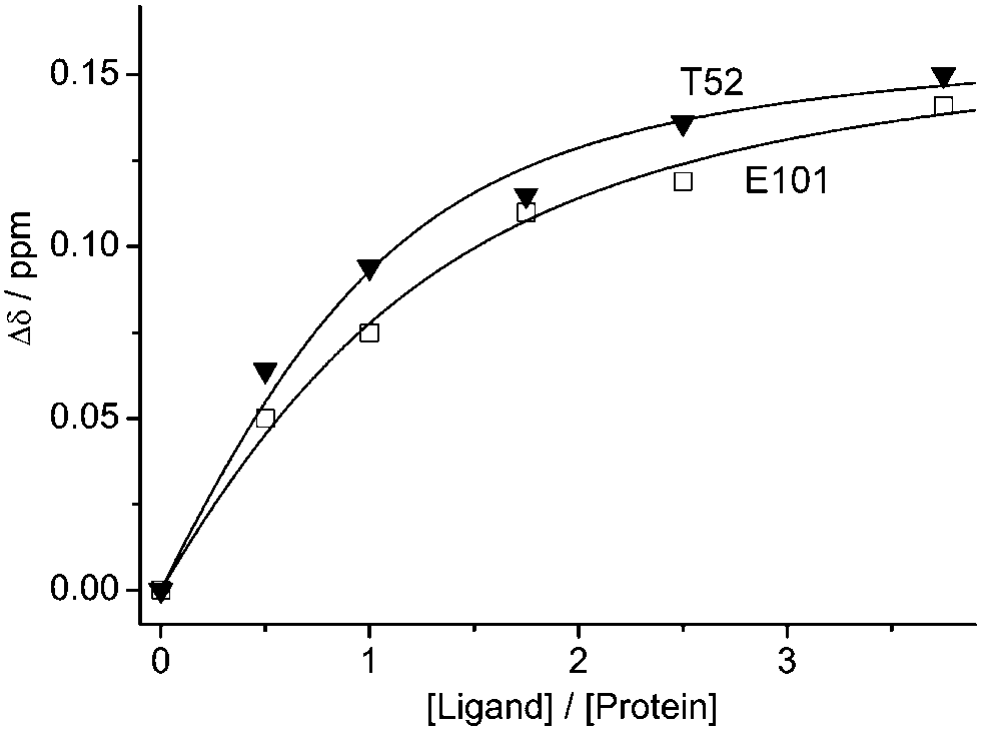
Binding affinity of compound 1 to WNV NS2B(K96A)-NS3pro. The changes in ^1^H chemical shifts of the backbone amide protons of Thr52 and Glu101 were plotted as a function of increasing concentration of compound 1. A 10 mM stock solution of compound 1 was titrated into a 80 *µ*M solution of ^15^N/^13^C-labeled NS2B(K96A)-NS3pro containing 20 mM phosphate (pH 7.0) and 2 mM DTT. The ^15^N-HSQC spectra were recorded at 25°C on an 800 MHz NMR spectrometer. The 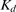 value derived from the fitting curves is about 40 *µ*M (30±10 and 50±10 *µ*M for the cross-peaks of Thr52 and Glu101, respectively).

The DAIM decomposition of the 11715 compound library yielded 1148 unique fragments. These 11715 compounds were docked by FFLD and the resulting poses of each compound were first clustered by using a leader algorithm with a similarity cutoff of 0.7 [50],[65]. The cluster representatives (a total of 265181 poses) were further minimized using CHARMM and the CHARMm force field with a distance-dependent dielectric function. Poses with less than three hydrogen bonds with the protein were filtered out. This filter is justified by the strong polar character of the S1–S3 pockets and the high number of hydrogen bonds observed between known inhibitors and the WNV protease, e.g., the aldehyde inhibitor Bz-nKRR-H (IC_50_ = 4 *µ*M) [13]. The number of hydrogen bonds formed between compound and protein was checked for each pose by CHARMM with a cutoff of 3.5 Å between donor and acceptor. The 45248 poses forming at least 3 hydrogen bonds with the protein were further selected and evaluated by LIECE (1902 unique molecules). Finally, the 1000 poses (354 unique compounds) with most favorable LIECE energies and the 1000 poses (297 unique compounds) with most intermolecular hydrogen bonds were selected for visual inspection (Figure 2). A total of 22 compounds were ordered and tested using NMR spectroscopy (see below). The 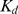 of compound **1** (Figure 3) is about 40 *µ*M according to ^1^H chemical shift (Figure 4). Notably, compound **1**, which has a durene (i.e., 1,2,4,5-tetramethylbenzene) scaffold linking two positively charged carbamimidothioate groups, has a ligand efficiency of 0.33 kcal/mol per non-hydrogen atom, which is good for further development (the threshold being at about 0.30 kcal/mol per non-hydrogen atom [66]).

### Validation by ^15^N-HSQC NMR spectra

**Figure 5:**
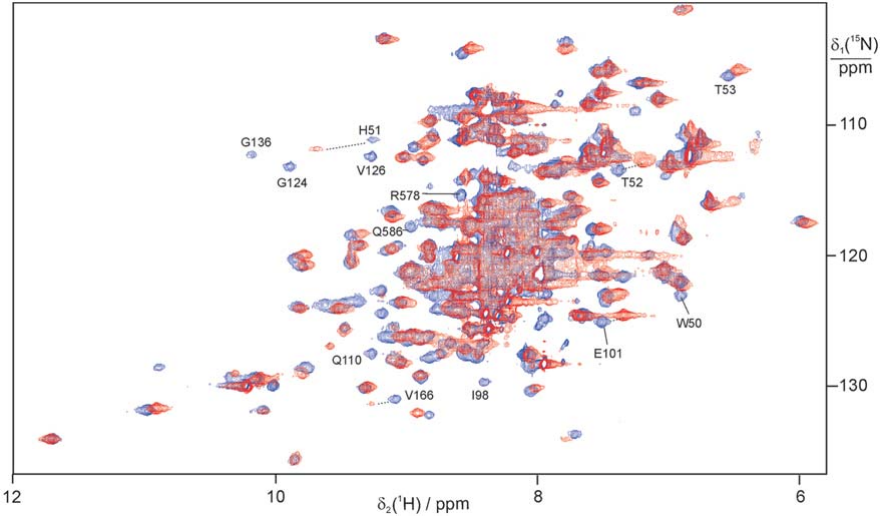
Superimposition of ^15^N-HSQC spectra of WNV NS2B(K96A)-NS3pro in the absence (red) and presence (blue) of compound 1 at 25°C. The resonance assignments are shown for selected peaks. The samples contained 0.9 mM protein in 90% H2O/10% D2O, 20 mM HEPES buffer (pH 7.0), 2 mM DTT. The complex with compound 1 was prepared by titrating the protein solution with a single addition of 15 *µ*l of a DMSO solution of compound 1 to a final concentration of 3 mM. The spectra were recorded at a ^1^H NMR frequency of 800 MHz.

The 22 compounds suggested by high-throughput docking were tested for binding to the WNV protease by NMR spectroscopy using ^15^N-labeled protein. 1D ^1^H NMR spectra were used to assess any line broadening experienced by the low-molecular weight compounds and ^15^N-HSQC spectra were recorded to detect responses in the protein. Twelve compounds showed broad lines in the presence of the WNV protease and six of them showed specific binding (Text S1). Among them, compound **1** significantly shifted the ^15^N-HSQC cross peaks of residues in the substrate binding site of the protein (Figure 5). The binding affinity of compound **1** was determined by measuring the change of ^1^H chemical shifts of the backbone amide protons of Thr52 and Glu101, which are located in the active site of the protein, as a function of increasing concentration of compound **1**. 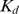 values of 30±10 *µ*M and 50±10 *µ*M were derived from the fits to the chemical shift changes of Thr52 and Glu101, respectively (Figure 4). To obtain a structure-activity relation, twelve commercially available compounds from Sigma-Aldrich and Maybridge Ltd containing carbamimidoylsulfanylmethyl as a substructure (Compounds **23**–**34**, see Text S1) were ordered and tested by NMR spectroscopy. However, all twelve compounds showed weaker binding affinities than **1**.

### Enzymatic assay and tryptophan fluorescence assay

The inhibitory activity of compound **1** was further confirmed by an enzymatic assay (IC_50_ = 178 *µ*M, ligand efficiency of 0.28 kcal/mol per non-hydrogen atom, Text S1) and a tryptophan fluorescence assay (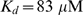, ligand efficiency of 0.31 kcal/mol per non-hydrogen atom, Text S1). The binding affinity measured by three different experimental techniques ranges from 40 *µ*M to 178 *µ*M (and −4.3 kcal/mol as predicted by the LIECE three-parameter model). These discrepancies might originate from the intrinsic differences in the mechanism of the three assays. The ^15^N-HSQC NMR spectra report directly on the binding of the compound to the protein, and the binding affinity is derived from the change of ^1^H chemical shifts of the backbone amide protons of Thr52 and Glu101. On the other hand, the enzymatic assay and tryptophan fluorescence assay are both competitive tests. The IC_50_ is derived in the enzymatic assay by monitoring the competitive binding of **1** and the (non-natural) substrate (Bz-nKRR-AMC), while the 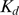 in the tryptophan fluorescence assay is obtained by competition with a noncovalent inhibitor (Text S1). Besides these differences other possible reasons of the discrepancies might be related to the different conditions of buffers, pH, etc. However, the specific binding of compound **1** is confirmed by NMR spectroscopy and its low affinity is probably due to its small size. Its ligand efficiency calculated using the values from the different techniques is always close to 0.30 kcal/mol per non-hydrogen atom, which supports the potential for further development [66]. In fact, the substrate binding site of the WNV protease is large and there are possibilities to improve the affinity of compound **1** by medicinal chemistry (see below).

### Binding mode and suggestions for further optimization

**Figure 6:**
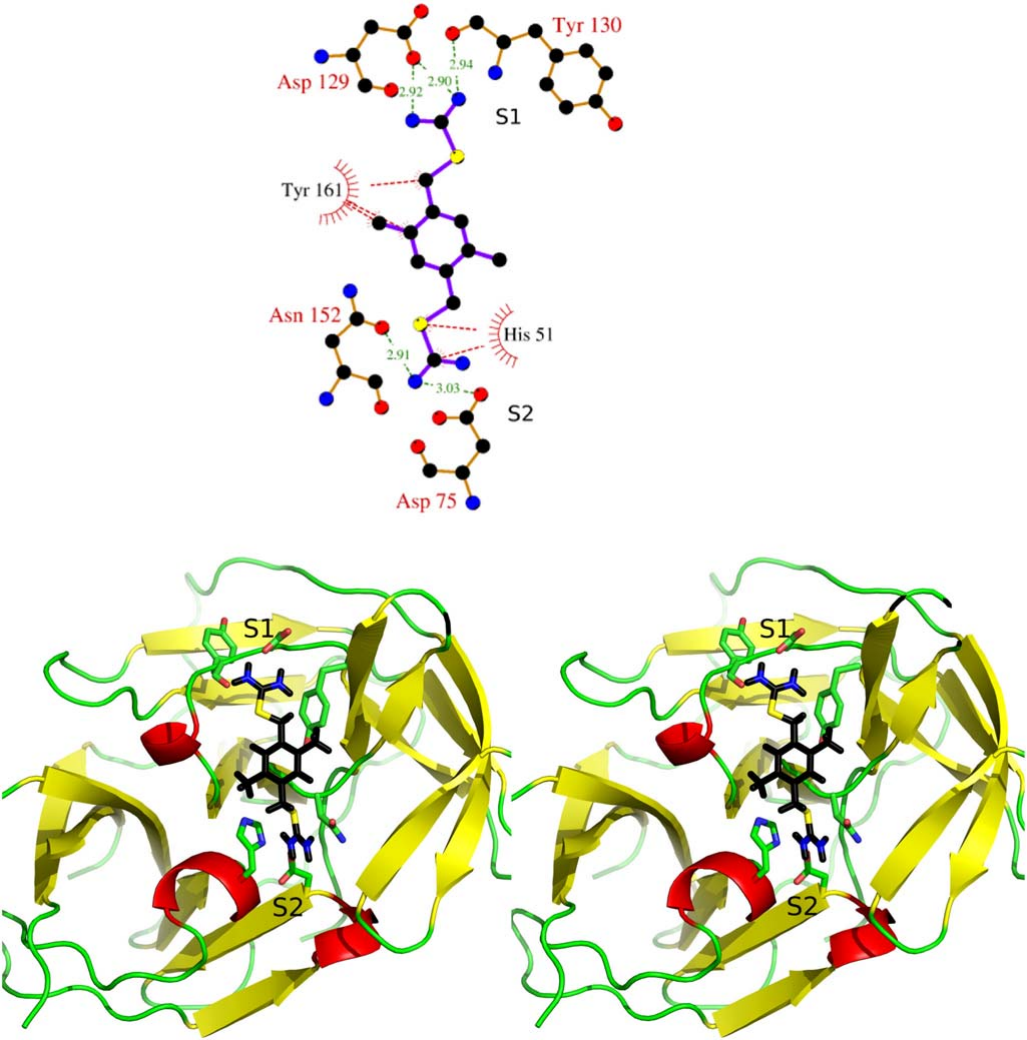
Binding mode of compound 1. (Top) Interactions between compound 1 and WNV protease. The atoms of the protein and ligand are shown as balls and colored as carbon in black, oxygen in red, nitrogen in blue, and sulfur in yellow. Intermolecular hydrogen bonds are shown by green dashed lines with distances in Å. The intermolecular van der Waals contacts interactions (distance ≤4 Å) are shown in red dashed lines. (Figure made by Ligplot [67]). (Bottom) Stereoview of the binding mode of 1. The protease is shown as cartoon and its secondary structure is emphasized by different colors: 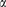 helices in red, 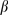 strands in yellow, and loops in green. Atoms of compound 1 are colored as in the (top) with hydrogens in black. This figure was prepared using PyMOL (Delano Scientific, San Carlos, CA).

The binding mode of compound **1** obtained by automatic docking with FFLD and CHARMM minimization (Figure 6) was confirmed by NMR spectroscopy [58]. In particular, both methyl groups and hydrogens of phenyl group (positions 1, 1′, 2, and 2′, see Figure 3) are within 5 Å from the side chains of His51, Thr132, Ile155, and Tyr161, which is in agreement with the intermolecular NOEs. The hydrophobic interactions between two methyl groups and protein side chains contribute to affinity as confirmed by the weaker binding (measured by NMR spectroscopy, Text S1) of compound **23** which is similar to **1** but lacks the two methyl groups.

In the predicted binding mode, there is a stacking interaction between the carbamimidothioate moiety and the imidazole ring of His51 in the S2 pocket. Furthermore, the two positively charged carbamimidothioate groups are involved in hydrogen bonds with several residues in the S1 and S2 pockets. The hydrogen bond acceptor groups in S1 are the Asp129 side chain and Tyr130 backbone carbonyl oxygen, and in S2 they are the Asn152 side chain as well as the NS2B Asp75 side chain. The lack of activity of two related compounds (**32** and **33**, Text S1) with two phenyl substituents linked to the terminal nitrogens is consistent with the binding mode of compound **1**.

**Figure 7:**
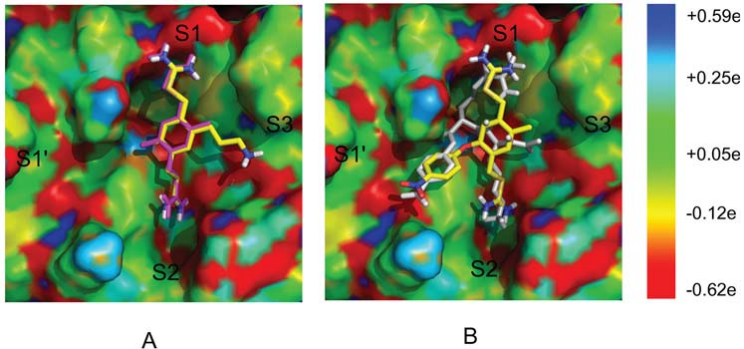
The protease surface (PDB code 2FP7) is colored according to atomic partial charges. (Left): Binding mode of the propylamine derivative of compound 1. Compound 1 and its propylamine derivative are in magenta and colored by atom type, respectively. (Right): Binding mode of the nitrophenyl derivative of compound 1. The substrate 2-naphthoyl-KKR-pNA [4] and the nitrophenyl derivative of compound 1 are in white and colored by atom type, respectively. The binding mode of the substrate is modelled based on Ref. 4 and only part of it is shown for clarity. These figures were prepared using PyMOL (Delano Scientific, San Carlos, CA).

As mentioned above, compound **1** is relatively small (18 heavy atoms) and occupies only two pockets (S1 and S2) of the substrate binding site. The binding mode obtained by docking and validated by NMR spectroscopy suggests that substitutions of one or both methyl groups of compound **1** might improve its binding affinity. In particular, replacing a methyl group with a propylamine might capture additional van der Waals and electrostatic interactions with residues in the S3 pocket (Figure 7(Left)). The LIECE free energy of binding of compound **1** and its propylamine derivative are −4.3 and −4.9 kcal/mol, respectively. Furthermore, replacement of a methyl group with a nitrophenyl-based (Figure 7(Right)) or coumarin-based substituent might yield favorable interactions with residues at the S1′ pocket. These modifications are inspired by the similar orientations of one of the two methyl groups of compound **1** and the C-terminal part of the aldehyde inhibitor Bz-nKRR-H, and the fact that nitrophenyl and coumarin are expected to bind to the S1′ pocket as they are linked at the C-terminus of the substrate 2-naphthoyl-lysine-lysine-arginine-para-nitroanilide (2-naphthoyl-KKR-pNA) [4] and Bz-nKRR-AMC (used in the enzymatic assay of this work), respectively.

### Conclusions

High-throughput, fragment-based docking into the active site of the WNV protease and binding free energy evaluation were used to select 22 compounds for experimental validation from an initial library of nearly 12000 molecules. Six of the 22 compounds showed specific affinity according to the NMR spectra of the compounds and the protein. Compound **1**, which had the most pronounced effects on the NMR spectrum of the protease, was further characterized and shown to bind specifically to the active site with an affinity of about 40 *µ*M (ligand efficiency of 0.33 kcal/mol per non-hydrogen atom) on the basis of changes of ^1^H chemical shifts of the backbone amide protons of two active site residues. The inhibitory activity of compound **1** was further validated in an enzymatic assay (IC_50_ = 183 *µ*M) and a tryptophan fluorescence assay (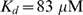). Finally, its predicted binding mode was confirmed by intermolecular NOEs and used to suggest three chemical modifications with the aim of improving the binding affinity.

### Availability of the Software

The software suite of programs for high-throughput docking (DAIM, SEED, FFLD), including input files, test cases and documentation, are available from the corresponding author (at no expenses for not-for-profit institutions).

## Supporting Information

Alternative Language Abstract S1 Translation of the Abstract into French by Christophe Bodenreider
(0.02 MB DOC)

Alternative Language Abstract S2 Translation of the Abstract into Polish by Dariusz Ekonomiuk
(0.02 MB PDF)

Alternative Language Abstract S3 Translation of the Abstract into German by Gottfried Otting
(0.07 MB DOC)

Alternative Language Abstract S4 Translation of the Abstract into Italian by Marino Convertino and Amedeo Caflisch
(0.02 MB DOC)

Alternative Language Abstract S5 Translation of the Abstract into Chinese by Danzhi Huang
(0.02 MB PDF)

Text S1 Supporting Information. Includes a list of 37 peptide aldehyde inhibitor sequences and their experimentally measured binding free energy used in deriving the LIECE models, enzymatic assay IC_50_ curve, tryptophan fluorescence assay *K_d_* curve, structures of 22 compounds and NMR spectroscopy results, structures of 12 compounds retrieved from substructural search and NMR spectroscopy results, structures of three suggested compounds.
(0.67 MB PDF)
