# Supplementary material for: Discovery of a Non-Peptidic Inhibitor of West Nile Virus NS3 Protease by High-Throughput Docking
Source: PLoS Negl Trop Dis. 2009 Jan 13;3(1):e356. doi: 10.1371/journal.pntd.0000356 (PMC2613028; doi:10.1371/journal.pntd.0000356)
Supplement: Alternative Language Abstract S1 — Translation of the Abstract into French by Christophe Bodenreider (0.02 MB DOC) [file pntd.0000356.s001.doc]

Chez les flavivirus tels que les virus du Nil occidental (WNV) et de la dengue, le domaine protéase de la protéine non structurale 3 (NS3pro) joue un rôle essentiel dans la réplication virale et constitue par conséquent une cible priviligiée pour le développement d’antiviraux. Nous rapportons ici l’identification d’une molécule inhibitrice de WNV NS3pro. Cela a été effectué par criblage in silico (fragment-based docking) d’une librairie comportant 12000 composés chimiques ainsi que par résonance magnétique nucléaire (RMN). La liaison spécifique de cet inhibiteur au site actif de WNV NS3pro a été confirmée par spectre RMN 15N-HSQC et par extinction de fluorescence des tryptophanes puis validée par test enzymatique. L’inhibiteur découvert a un bon rapport affinité-poids moléclaire. En effet l’efficacité du ligand représente 0.33kcal/mol par atome en excluant les atomes d’hydrogène. Il constitue donc un composé à partir duquel des antiviraux efficaces contre le virus du Nil occidental pourront être développés.
