# Supplementary material for: Discovery of a Non-Peptidic Inhibitor of West Nile Virus NS3 Protease by High-Throughput Docking
Source: PLoS Negl Trop Dis. 2009 Jan 13;3(1):e356. doi: 10.1371/journal.pntd.0000356 (PMC2613028; doi:10.1371/journal.pntd.0000356)
Supplement: Alternative Language Abstract S2 — Translation of the Abstract into Polish by Dariusz Ekonomiuk (0.02 MB PDF) [file pntd.0000356.s002.pdf]

Translation of the abstract into Polish by Dariusz Ekonomiuk

Discovery of a non-peptidic inhibitor of West Nile virus NS3 protease by high-throughput docking.

Odkrycie niepeptydowego inhibitora proteazy NS3 wirusa Zachodniego Nilu przy wykorzystaniu wirtualnego screeningu.

5 listopada 2008

Streszczenie

Niestrukturalna proteaza NS3pro jest kluczowym enzymem Flawiwirusów i w związku z tym jest bardzo ważnym celem w projektowaniu leków przeciwko zakażeniom wirusem Zachodniego Nilu oraz wirusami Dengi.

Inhibitor proteazy WNV NS3pro (charakteryzujący się niewielką masą cząsteczkową) został zidentyfikowany przy pomocy automatycznego dokowania opartego na fragmentach (ang. automatic fragment-based docking). Z początkowej biblioteki związków składającej się z 12000 substancji tylko 22 zostały wybrane do dalszego testowania z wykorzystaniem magnetycznego rezonansu jądrowego (NMR). Otrzymane widma ^{15}N-HSQC NMR potwierdziły zarówno specyficzny charakter oddziaływania jak i teoretycznie przewidzianą konformację inhibitora oddziałującego z aminokwasami w miejscu aktywnym proteazy.

Zidentyfikowany inhibitor charakteryzuje się dobrym iloczynem energii oddziaływania względem masy molekularnej i w związku z tym jest dobrym kandydatem na związek wiodący w procesie rozwoju leków skierowanym przeciwko zakażeniu wirusem Zachodniego Nilu.
