# Supplementary material for: Discovery of a Non-Peptidic Inhibitor of West Nile Virus NS3 Protease by High-Throughput Docking
Source: PLoS Negl Trop Dis. 2009 Jan 13;3(1):e356. doi: 10.1371/journal.pntd.0000356 (PMC2613028; doi:10.1371/journal.pntd.0000356)
Supplement: Alternative Language Abstract S3 — Translation of the Abstract into German by Gottfried Otting (0.07 MB DOC) [file pntd.0000356.s003.doc]

Automatisches Docking von 12000 Verbindungen gefolgt von Tests von nur 22 Molekülen mit kernmagnetischer Resonanzspektroskopie (NMR) führte zur Identifizierung eines mikromolaren Inhibitors der nicht-strukturellen Westnilvirus-Protease 3 (NS3pro). Die Spezifizität der Bindung am aktiven Zentrum des NS3pro Enzyms wurde durch ^15^N-HSQC-NMR-Spektren nachgewiesen. Der Inhibitor, [4-carbamimidoylsulfanylmethyl)-2,5-dimethylphenyl]methylsulfanylmethanimidamide, weist ein günstiges Verhältnis von Bindungsaffinität zu Molekülgewicht auf mit einer Ligandeneffizienz von 0.33 kcal/mol pro Nichtwasserstoffatom, und ist somit eine potentielle Leitverbindung.
