# Supplementary material for: Discovery of a Non-Peptidic Inhibitor of West Nile Virus NS3 Protease by High-Throughput Docking
Source: PLoS Negl Trop Dis. 2009 Jan 13;3(1):e356. doi: 10.1371/journal.pntd.0000356 (PMC2613028; doi:10.1371/journal.pntd.0000356)
Supplement: Alternative Language Abstract S4 — Translation of the Abstract into Italian by Marino Convertino and Amedeo Caflisch (0.02 MB DOC) [file pntd.0000356.s004.doc]

La proteasi non-strutturale NS3pro è un enzima essenziale dei flavivirus e quindi costituisce un bersaglio promettente per sviluppare farmaci contro il West Nile virus e la febbre Dengue. Abbiamo identificato un inibitore della citata proteasi tramite docking di circa 12000 composti e validazione sperimentale di 22 molecole a mezzo di risonanza magnetica nucleare (RMN). Il legame specifico dell'inibitore nel sito attivo è stato verificato tramite spettri RMN di tipo 15N-HSQC. La sua attivita` inibente e` stata validata ulteriormente utilizzando un saggio enzimatico ed un saggio di fluorescenza (basato sul triptofano). L'inibitore [4-(carbamimidoilsulfanilmetil)-2,5-dimetilfenil]-metilsulfanilmetanimidamide presenta un buon quoziente affinità/peso molecolare ("ligand efficiency" di 0.33 kcal/mol per atomo pesante) ed ha quindi un buon potenziale come composto "lead" per un ulteriore sviluppo allo scopo di combattere le infezioni da West Nile virus.
