# Supplementary material for: Discovery of a Non-Peptidic Inhibitor of West Nile Virus NS3 Protease by High-Throughput Docking
Source: PLoS Negl Trop Dis. 2009 Jan 13;3(1):e356. doi: 10.1371/journal.pntd.0000356 (PMC2613028; doi:10.1371/journal.pntd.0000356)
Supplement: Alternative Language Abstract S5 — Translation of the Abstract into Chinese by Danzhi Huang (0.02 MB PDF) [file pntd.0000356.s005.pdf]

非结构性蛋白酶 3 是黄病毒的一种基本的蛋白酶， 所以它是一个非常有应用前景的西尼罗河病毒和登革热病毒的药物靶蛋白。在这个工作中，我们从 12000 个小分子中用了高通量筛选和核磁检测的方法从 22 个小分子找到了一个西尼罗河病毒非结构性蛋白酶 3 的小分子抑制剂。这个抑制剂的特异性以及结合的方式也用核磁的方法确认了。我们还用了另外的两种实验方法，酶活测试和色氨酸荧光熄灭法验证了这一结果。这个小分子具有非常好的抑制效率（0.33 千卡每摩尔每个重原子），说明了它是一种非常有潜力的治疗西尼罗河病毒的先导分子。
