# Supplementary material for: Discovery of a Non-Peptidic Inhibitor of West Nile Virus NS3 Protease by High-Throughput Docking
Source: PLoS Negl Trop Dis. 2009 Jan 13;3(1):e356. doi: 10.1371/journal.pntd.0000356 (PMC2613028; doi:10.1371/journal.pntd.0000356)

# Supporting Information

## Discovery of a non-peptidic inhibitor of West Nile virus NS3 protease by high-throughput docking

Dariusz Ekonomiuk[a], Xun-Cheng Su[b], Kiyoshi Ozawa[b], Christophe Bodenreider[c], Siew Pheng Lim[c], Zheng Yin[c], Thomas H. Keller[c], David Beer[c], Viral Patel[c], Gottfried Otting[b], Amedeo Caflisch[a], and Danzhi Huang[a*]

[a] Department of Biochemistry
University of Zürich, Winterthurerstrasse 190
CH-8057 Zürich, Switzerland
Phone: (+41 44) 635 55 68, FAX: (+41 44) 635 68 62
email: dhuang@bioc.uzh.ch

[b] Research School of Chemistry
The Australian National University
Canberra ACT 0200, Australia

[c] Novartis Institute for Tropical Diseases Pte Ltd
Biopolis Road 10
05-01, Chromos, Singapore

* Corresponding author

Keywords: West Nile virus, high-throughput docking, fragment-based lead identification, NMR spectroscopy

November 5, 2008

# Contents


1 **Structures and affinities of 37 known inhibitors** **S-3**

2 **Binding studies by NMR spectroscopy** **S-4**

3 **Enzymatic assay $IC_{50}$ curve** **S-7**

4 **Tryptophan fluorescence assay $K_d$ curve** **S-8**

5 **Structures of 22 compounds from docking** **S-9**

6 **Structures of 12 compounds from substructural search** **S-17**

7 **Structures of 3 suggested compounds** **S-18**

# 1 Structures and affinities of 37 known inhibitors

Table S-I: Peptide aldehyde inhibitors of WNV NS2B-NS3pro and binding affinity measured as in Knox et al., J. Med. Chem. **49**, 6585, 2006[a]

| ID | Structure | no. of formal charges | $IC_{50}$ ($\mu$M) | ΔG (kcal/mol) |
|---|---|---|---|---|
| 1 | Bz-Nle-Lys-Arg-Arg-H | 3 | 4.1 | -7.39 |
| 2 | Bz-Nle-Lys-Lys(Z)-Arg-H | 2 | 99.5 | -5.49 |
| 3 | Bz-Nle-Lys-Gln-Arg-H | 2 | 1.7 | -7.9 |
| 4 | Bz-Nle-Lys-Lys-Arg-H | 3 | 1.9 | -7.86 |
| 5 | Ac-Lys-Lys-Arg-H | 3 | **0.4** | -8.84 |
| 6 | Bz-Lys-Arg-Arg-H | 3 | 1.5 | -7.99 |
| 7 | Bz-Lys-Lys(Tos)-Arg-H | 2 | 117.9 | -5.39 |
| 8 | Ac-Lys-Lys(Tos)-Arg-H | 2 | **463.4** | -4.58 |
| 9 | Ac-Lys-Lys(Bz)-Arg-H | 2 | 116.5 | -5.4 |
| 10 | Bz-Lys-(p-Me)Phe-Arg-H | 2 | 194.3 | -5.09 |
| 11 | Bz-Lys-Lys(Bz)-Arg-H | 2 | 68.1 | -5.72 |
| 12 | indole-Lys-Arg-Arg-H | 3 | 2.4 | -7.72 |
| 13 | Bz-Lys-Asn-Arg-H | 2 | 71.8 | -5.69 |
| 14 | Bz-Nle-Ala-Arg-Arg-H | 2 | 3.8 | -7.44 |
| 15 | Bz-Ala-Lys-Arg-Arg-H | 3 | 0.7 | -8.45 |
| 16 | Bz-Nle-Lys-Arg-Phe-H | 2 | 109.8 | -5.43 |
| 17 | Bz-Nle-Lys-Phe-Arg-H | 2 | 108 | -5.44 |
| 18 | Bz-Nle-Phe-Arg-Arg-H | 2 | 4.2 | -7.38 |
| 19 | Bz-Phe-Lys-Arg-Arg-H | 3 | 1.2 | -8.14 |
| 20 | Bz-Lys-Arg-Tyr-H | 2 | 14.6 | -6.64 |
| 21 | Ac-Lys-Arg-Arg-H | 3 | 0.5 | -8.6 |
| 22 | pyridine-Lys-Arg-Arg-H | 3 | 0.8 | -8.4 |
| 23 | isoquinoline-Lys-Arg-Arg-H | 3 | 0.6 | -8.56 |
| 24 | pyrazine-Lys-Arg-Arg-H | 3 | 1.1 | -8.18 |
| 25 | 3-pyridyl-Lys-Arg-Arg-H | 3 | 1 | -8.24 |
| 26 | Bzl-Nle-Lys-Arg-(4-CN)-Phe-H | 2 | 62 | -5.77 |
| 27 | Bzl-Nle-Lys-Arg-Trp-H | 2 | 10 | -6.86 |
| 28 | Bz-Nle-Lys-Arg-Lys-H | 3 | 57.7 | -5.82 |
| 29 | BZ-Nle-Lys-Arg-(4-guanidinyl)-Phe-H | 3 | 11.8 | -6.76 |
| 30 | Bz-Nle-Lys-Arg-His-H | 2 | 43.1 | -5.99 |
| 31 | Bz-Nle-Lys-Arg-Phg-H | 2 | 90.9 | -5.55 |
| 32 | Bz-Arg-Arg-H | 2 | 3.9 | -7.42 |
| 33 | Bz-Lys-Arg-H | 2 | 1.4 | -8.03 |
| 34 | Bz-Arg-Lys-H | 2 | 57.5 | -5.82 |
| 35 | Bz-Lys-Arg-Phe-H | 2 | 71.1 | -5.69 |
| 36 | Bz-Lys-Arg-(P-Me)Phe-H | 2 | 17.7 | -6.52 |
| 37 | Bz-Lys-Arg-Tyr(Bn)-H | 2 | 11.8 | -6.76 |

[a] Weakest and strongest affinities are in bold.

# 2 Binding studies by NMR spectroscopy

The binding of compounds to the WNV NS2B-NS3 protease (henceforth called protease) was assessed by NMR spectroscopy using 50 mM stock solutions of the compounds prepared by dissolution in DMSO-$d_6$. For each compound, the following NMR spectra were measured at 800 MHz and 25 °C.

1) A 1D $^1$H NMR spectrum in $D_2O$ to check the spectral appearance of the compound and to assess its aggregation state (narrow signals are expected for the monomeric state). The samples were prepared by adding 1 μl of stock solution to 200 μl $D_2O$, resulting in a compound concentration of about 0.25 mM.

2) A 1D $^1$H NMR spectrum of $^{15}$N-labeled protease in the presence of an about 3-fold excess of compound. The samples were prepared by adding 3 μl of stock solution to 200 μl of a 0.23 mM solution of protease in 90% $H_2O$/10% $D_2O$, 20 mM HEPES buffer, pH 7.0, 2 mM DTT. Any compound binding to the protease displayed broad signals because (i) transverse relaxation is faster in the complex with the macromolecule and (ii) chemical exchange between bound and free compound results in exchange broadening. In the limit of slow exchange between bound and free compound, narrow signals would be expected for the excess of free compound. This case was not observed in the present study, as compounds displaying narrow signals in the presence of the protease failed to change the $^{15}$N-HSQC spectrum of the protease (see below).

3) A 2D $^{15}$N-HSQC spectrum of the $^{15}$N-labeled protease in the presence of compound using the sample described above. Binding of a compound was classified as non-specific, if the spectrum of the compound (spectrum 2) displayed line broadening but no chemical shift changes could be detected in the $^{15}$N-HSQC spectrum. Binding of the compound was classified as very weak, if spectrum 2 displayed line broadening and any chemical shift changes in the $^{15}$N-HSQC spectrum were small (<0.03 ppm in the $^1$H dimension and <0.15 ppm in the $^{15}$N dimension). Notably, some of these changes could have arisen from small pH changes caused by some to the compounds. Changes in pH were also evidenced by small chemical shift changes of the signals of small amounts of residual imidazol present in the samples that originated from the purification protocol of the His-tagged protease.

Only compounds **1** and **2** changed the appearance of the $^{15}$N-HSQC spectrum substantially, indicating significant binding affinity and specific binding. The $^{15}$N-HSQC cross peaks of the NS3 residues T52, T53, E101, and K104 (at 112.7/7.22, 105.8/6.50, 124.4/7.38, and 118.0/7.54 ppm, respectively) were well resolved in the spectrum of the apo-protein and their chemical shifts relatively sensitive to the presence of compounds.

Table S-II: Binding of compounds assessed by NMR spectroscopy of 22 compounds

| Compound number | Broadening of the compound signals in spectrum 2 | Chemical shift changes in the $^{15}$N-HSQC spectrum | Binding |
|---|---|---|---|
| 1 | yes[1] | significant | yes |
| 2 | yes[1] | significant | yes |
| 3 | yes[1] | no | non-specific |
| 4 | yes | very small | very weak |
| 5 | no | no | no |
| 6 | yes[1] | no | non-specific |
| 7 | no | no | no |
| 8 | yes | very small | very weak |
| 9 | yes[1] | no | non-specific |
| 10 | no | very small[2] | no |
| 11 | no | no | no |
| 12 | no | no | no |
| 13 | no | no | no |
| 14 | no | no | no |
| 15 | no | no | no |
| 16 | no | very small[2] | no |
| 17 | no | no | no |
| 18 | yes | very small | very weak |
| 19 | yes[1] | significant but small | weak |
| 20 | yes[1] | no | non-specific |
| 21[3] | yes[1] | no | at most very weak |
| 22 | yes[1] | no | non-specific |

[1] broadened beyond detection

[2] possibly due to pH change

[3] barely soluble in water

Table S-III: NMR analysis of 12 compounds identified by substructure search

| Compound number | Broadening of the compound signals in spectrum 2 | Chemical shift changes in the $^{15}$N-HSQC spectrum | Binding |
|---|---|---|---|
| 23 | yes | very small | very weak |
| 24 | yes | very small | very weak |
| 25 | no | no | no |
| 26 | yes | no | no |
| 27 | yes | no | no |
| 28 | yes | no | no |
| 29 | yes | no | no |
| 30 | yes | no | no |
| 31 | no | no | no |
| 32 | yes | very small | very week |
| 33 | yes | no | no |
| 34 | yes | very small | very weak |

# 3 Enzymatic assay $IC_{50}$ curve

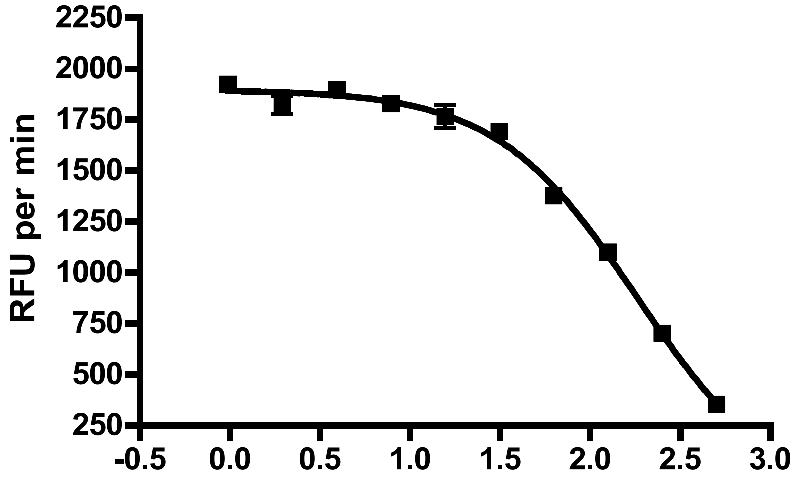


Log compound 1 (µM)

$IC_{50}$ of compound **1** was measured as described in Knox et al., J. Med. Chem. 49 **6585**,2006. The initial velocities in the enzyme reaction in the presence of increasing concentration of compound **1** (from 0 to 500 mM) against control wells without compounds were calculated and plotted. $IC_{50}$ value was derived by fitting the calculated initial velocities to a non linear regression curve using GraphPad Prism software. Each point of the $IC_{50}$ curve was carried out in duplicate during a single experiment.

# 4 Tryptophan fluorescence assay $K_d$ curve

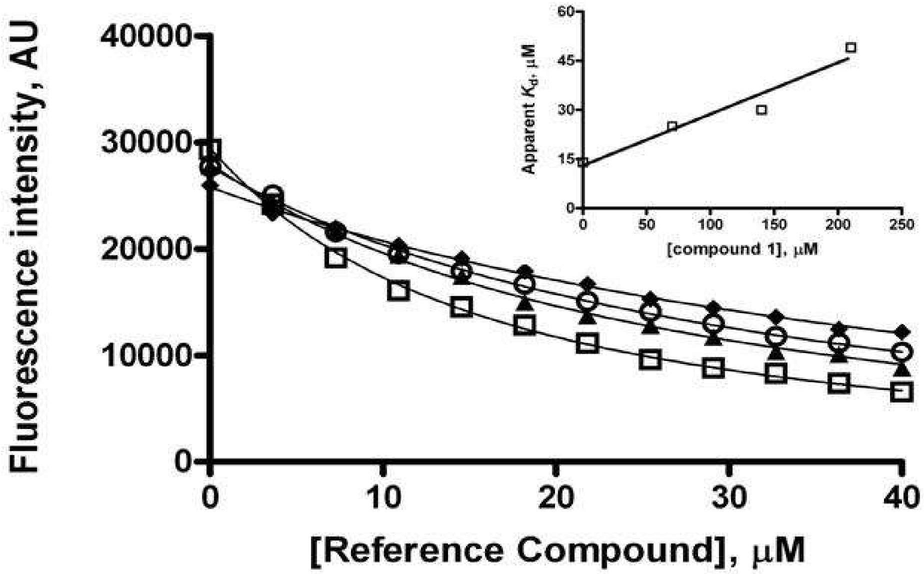


1

Binding affinity of compound **1** was measured by tryptophan fluorescence assay [Zhang, Y. L.; Zhang, Z. Y., *Anal Biochem* **1998,** 261, (2), 139-48.), Bodenreider C and et al., Manuscript to be submitted.]. Fluorescence titrations performed with a reference compound in presence of varying concentrations of compound **1** (0 μM (□), 70 μM (▲), 140 μM (○) and 240 μM (◆)). Solid lines show the fit of the data to a 1:1 binding equation. The resulting apparent $K_d$ values of the reference compound are reported as a function of compound **1** concentration in the insert. This dependence yields $K_d$ = 83 μM for binding of compound **1** to WNV protease. The reference compound is N,N-Bis-(4-(4-methyl-4,5-dihydro-1H-imidazol-2-yl-phenyl)-phthalazine-1,4-diamine.

# 5 Structures of 22 compounds from docking

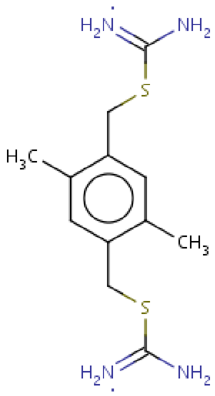


1

---

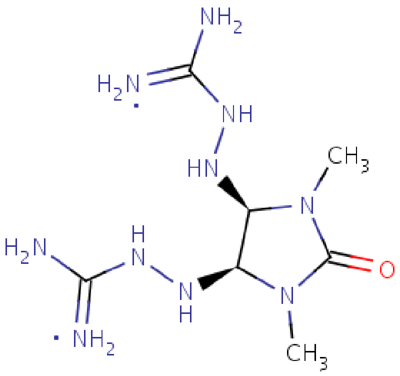


2

---

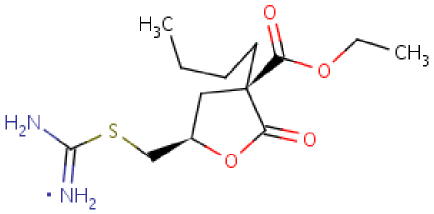


3

---

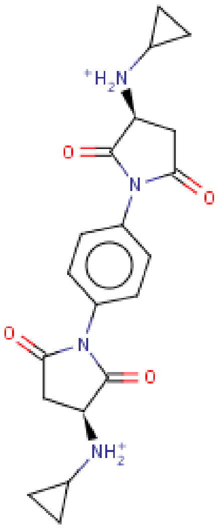
+H2N
O
N
O
O
N
O
NH2+

4

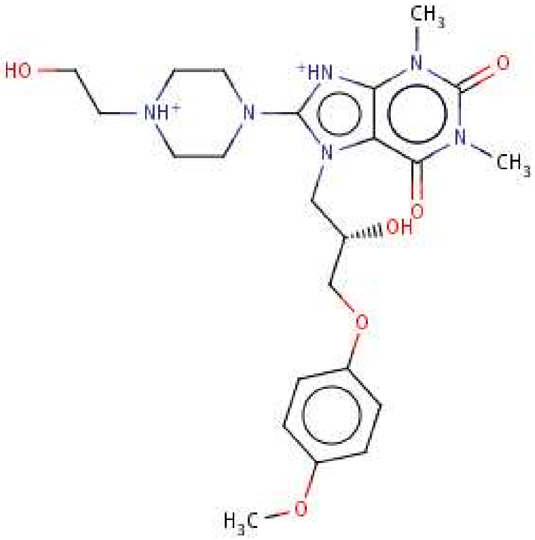
CH3
HO
NH+
N
+HN
N
O
N
CH3
N
O
OH
O
H3C
O

5

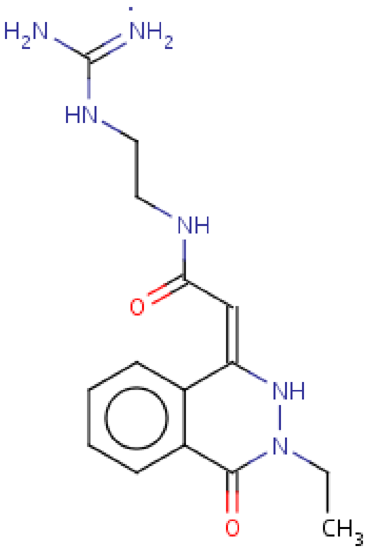
H2N
NH2
HN
NH
O
NH
N
O
CH3

6

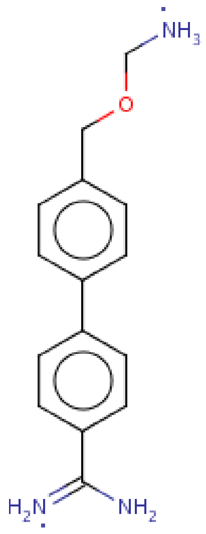


7

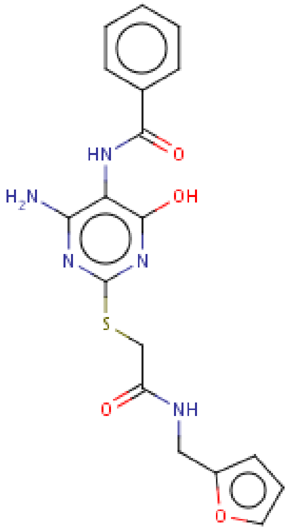


8

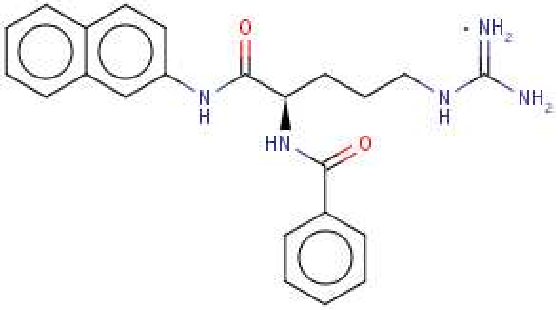


9

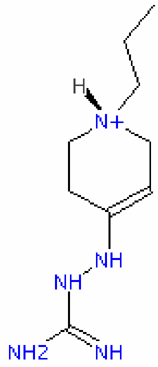


10

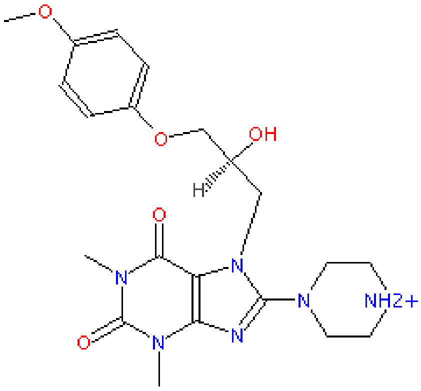


11

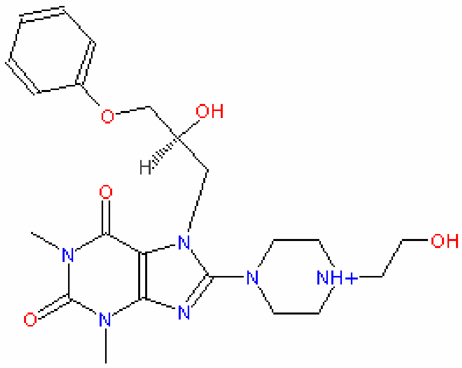


12

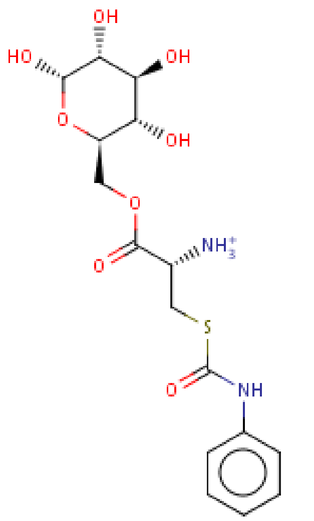
OH
HO
OH
O
OH
O
NH3+
O
S
O
NH

13

---

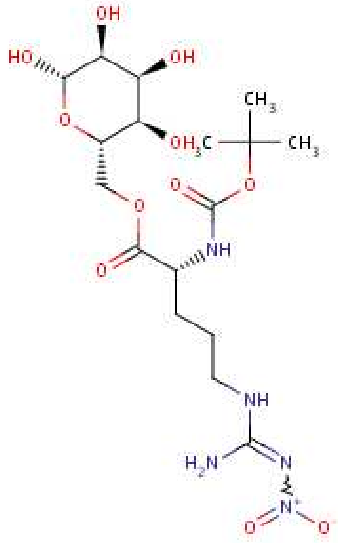
OH
HO
OH
CH3
O
OH H3C
CH3
O
O
O
NH
O
NH
H2N
N
N+
O
O-

14

---

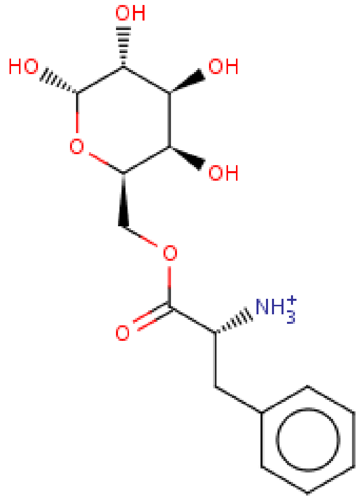
OH
HO
OH
O
OH
O
NH3+
O

15

---

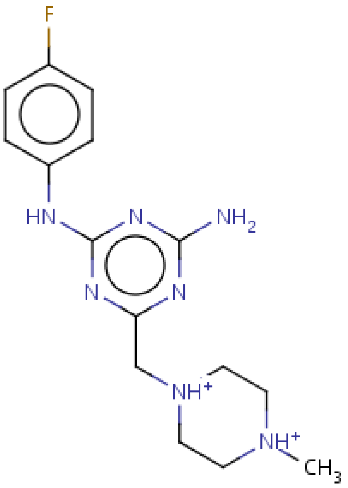
F
HN
N
NH2
N
N
NH+
NH+
CH3

16

---

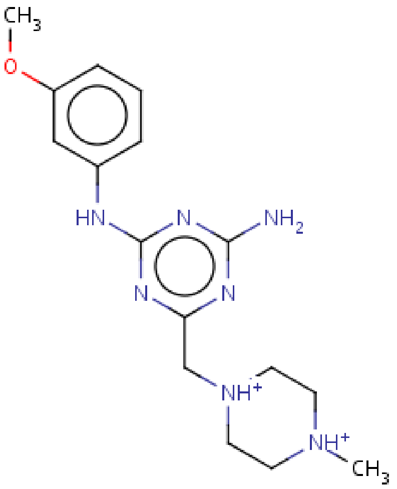
CH3
O
HN
N
NH2
N
N
NH+
NH+
CH3

17

---

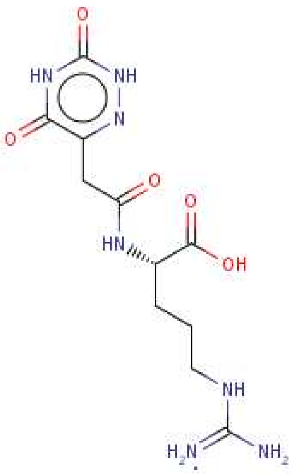
O
HN
NH
N
O
O
O
HN
OH
NH
H2N
NH2

18

---

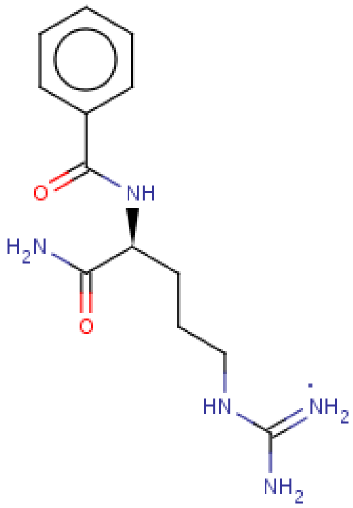
O
NH
H2N
O
HN
NH2
NH2

19

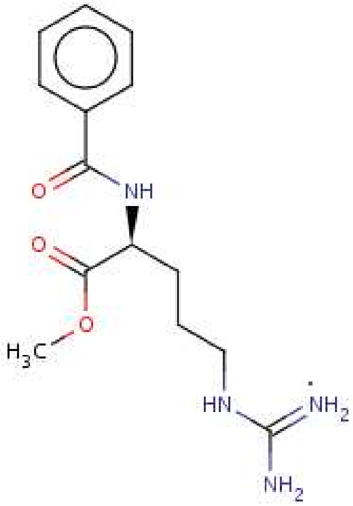
O
NH
O
O
H3C
HN
NH2
NH2

20

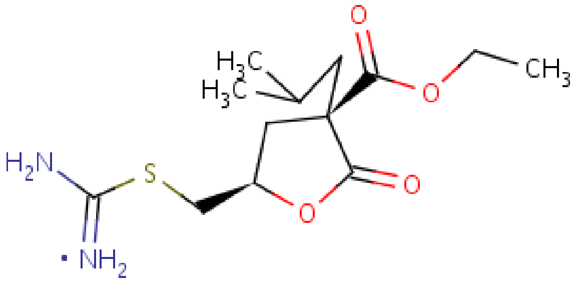
O
H3C
H3C
O
CH3
H2N
S
O
O
NH2

21

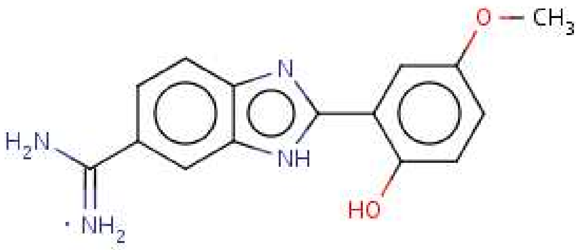


22

# 6 Structures of 12 compounds from substructural search

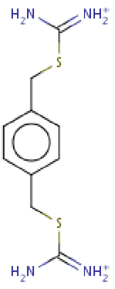

23

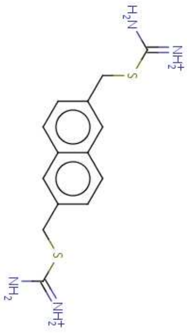

24

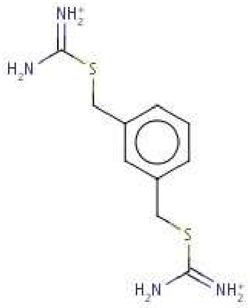

25

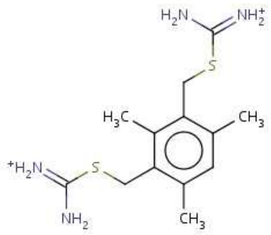

26

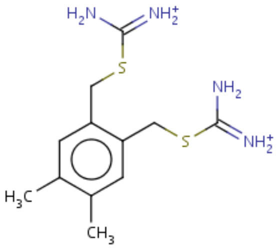

27

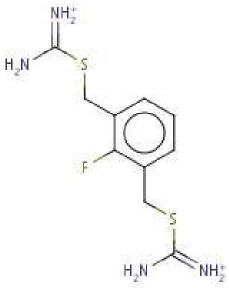

28

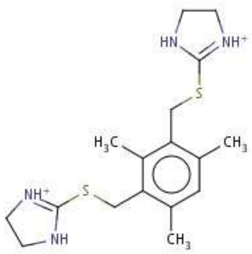

29

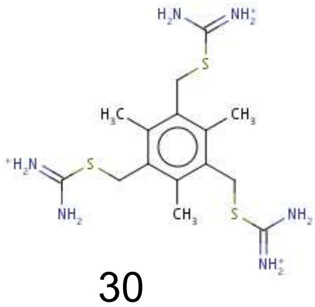

30

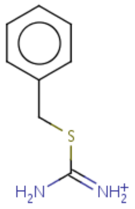

31

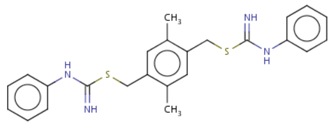

32

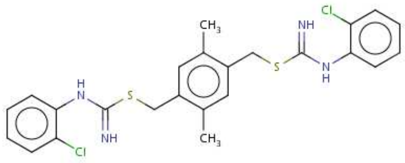

33

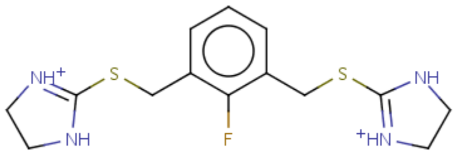

34

# 7 Structures of 3 suggested compounds

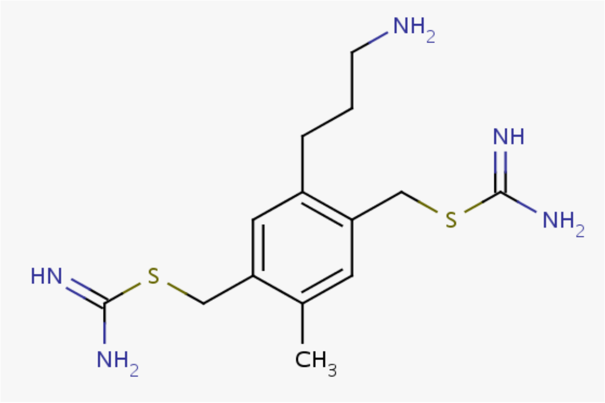


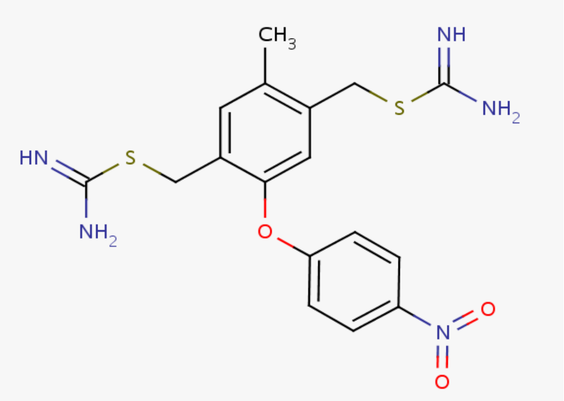


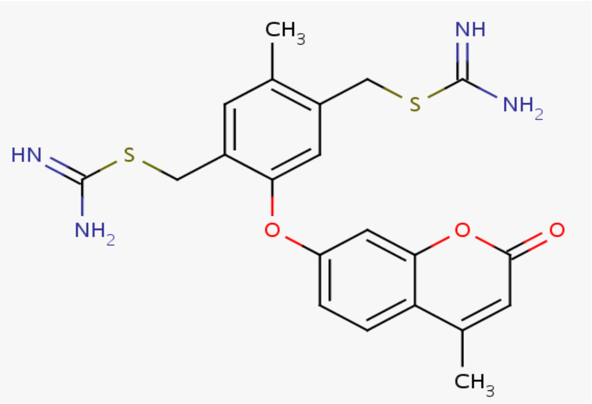

Supplement: Text S1 — Supporting Information. Includes a list of 37 peptide aldehyde inhibitor sequences and their experimentally measured binding free energy used in deriving the LIECE models, enzymatic assay IC50 curve, tryptophan fluorescence assay Kd curve, structures of 22 compounds and NMR spectroscopy results, structures of 12 compounds retrieved from substructural search and NMR spectroscopy results, structures of three suggested compounds. (0.67 MB PDF) [file pntd.0000356.s006.pdf]
